# Perceptions of mental disorder causes, treatments, and prevention among the general population in Saudi Arabia

**DOI:** 10.3389/fpsyt.2024.1404957

**Published:** 2024-06-24

**Authors:** Mohammad Senitan, Abdulhameed Abdullah Alhabeeb, Nora A. Althumairi, Mohammed M. J. Alqahtani, Rashed Abdullah Al-Duraihem, Nasser F. BinDhim

**Affiliations:** ^1^ Department of Public Health, College of Health Sciences, Saudi Electronic University, Riyadh, Saudi Arabia; ^2^ National Centre for Mental Health Promotion, Riyadh, Saudi Arabia; ^3^ Informed Decision-Making Research and Studies, Riyadh, Saudi Arabia; ^4^ Department of Psychology, King Khalid University, Abha, Saudi Arabia

**Keywords:** mental health perceptions, causes of mental disorders, treatment of mental disorders, mental health prevention, general population, Saudi Arabia

## Abstract

This study investigates the community’s perception of mental health in Saudi Arabia, emphasizing its influence on attitudes toward the causes, treatment, and prevention of mental health issues. This understanding is vital for creating evidence-based mental health interventions. A cross-sectional national mental health screening was conducted in 2023, utilizing proportional quota sampling for age, gender, and regional representation among 4547 Saudi participants aged 18–90, randomly selected from national databases. Data were gathered using the ZDataCloud system. The study covered all 13 administrative regions of Saudi Arabia: Riyadh, Makkah, Madinah, Qassim, Eastern Province, Asir, Tabuk, Hail, Northern Borders, Jazan, Najran, Baha, and Al-Jouf. The majority (86.5%) had no personal history of mental health diagnosis or treatment. Key findings identified psychological trauma (78.36%) and substance abuse (72.88%) as primary perceived causes of mental disorders. Preferred treatments included non-pharmacological psychotherapies (74.97%) and pharmacological methods (71.08%). Prevention strategies focused on raising awareness of mental illness (80.4%) and enhancing positive relationships (70.6%). A multivariate logistic regression analysis revealed significant associations between demographic variables and perceptions of mental disorder causes, treatments, and prevention strategies. Females were more likely than males to perceive psychological trauma, drug and alcohol abuse, and domestic violence and sexual harassment as causes of mental disorders. Regarding treatments, females and those with personal or close connections to mental health issues were more likely to endorse non-pharmacological psychotherapy and Roquia in the Qur’an. For prevention strategies, females, those with a mental health diagnosis, and those living with someone diagnosed with a mental disorder were more likely to endorse increasing awareness, positive social relationships, and physical activity. The study suggests integrating religious and social beliefs into mental health programs to enhance community engagement and effectiveness.

## 1 Introduction

Mental health, a crucial component of public health, is significantly influenced by cultural and societal beliefs. In Saudi Arabia, a unique blend of religious, social, and cultural factors shapes public perceptions of mental disorders (1). These perceptions not only influence the understanding of mental disorders but also affect how they are approached in terms of treatment and prevention. The societal stigma associated with mental illnesses often leads to reluctance in seeking professional help and a preference for traditional healing practices (2, 3).

In many Middle Eastern cultures, including Saudi Arabia, mental health issues are often viewed through a religious lens, leading to attributions of mental illness to spiritual or supernatural causes. This cultural perspective can significantly affect the acceptance and utilization of modern psychiatric treatments (4). Understanding these cultural nuances is essential for effective mental health service delivery.

The literature on mental health perceptions in Saudi Arabia and the broader Middle Eastern region highlights a complex interplay of cultural, religious, and social factors. Studies by Koenig et al. (5) and Almutairi (6) underscore the profound impact of Islamic beliefs on mental health perceptions, where mental disorders are often attributed to spiritual or supernatural causes, such as jinn possession or the evil eye. This spiritual interpretation can hinder the acceptance and utilization of psychiatric services, favoring traditional healing methods instead. Al-Krenawi and Graham (7) found that these cultural beliefs significantly influence help-seeking behaviors, with many individuals opting for religious leaders over mental health professionals (5–7).

Furthermore, research by Dardas and Simmons (8) in Jordan and by Al-Adawi et al. (9) in Oman reveals similar patterns, where societal stigma and misconceptions about mental illnesses prevail, leading to delays in seeking treatment and a reliance on non-medical interventions. A study by Qureshi et al. (10) in Saudi Arabia specifically highlights the lack of awareness and prevalent misconceptions about mental health among the general population, contributing to negative attitudes and stigma. These studies collectively emphasize the need for culturally sensitive mental health strategies that incorporate an understanding of local beliefs and practices. Addressing these cultural nuances is essential for developing effective public health policies, designing appropriate mental health services, and implementing educational campaigns aimed at reducing stigma and improving mental health literacy in the region (8–10).

Despite the importance of cultural context in mental health, there is a noticeable gap in comprehensive research exploring the general population’s views on mental disorders in Saudi Arabia. While some studies have focused on specific demographic groups, such as healthcare professionals or patients, the broader public’s perspectives remain underexplored (6). Existing literature suggests a lack of awareness and misconceptions about mental disorders among the general population in Saudi Arabia. These misconceptions could contribute to delays in seeking care and a preference for non-medical interventions (11).

To thoroughly understand mental health perceptions in Saudi Arabia, it is crucial to consider the cultural and religious nuances that shape these views. Deeply rooted in Islamic traditions, Saudi society often interprets mental health through a religious lens. Al-Adawi et al. (9) note that mental illnesses are frequently attributed to supernatural causes like possession by jinn or the evil eye, leading individuals to seek help from religious healers rather than mental health professionals. This cultural perspective affects the acceptance of psychiatric conditions and influences treatment preferences, favoring traditional and religious practices. Additionally, Al-Krenawi and Graham (7) highlight that stigma surrounding mental illness is prevalent in the Middle East, including Saudi Arabia, causing delayed treatment and marginalization. Understanding these contexts is vital for developing culturally sensitive and effective mental health strategies (5, 7, 9).

The rapidly evolving socio-economic landscape of Saudi Arabia, marked by sweeping changes under initiatives like Vision 2030, further necessitates an updated understanding of public perceptions of mental health. Vision 2030 is a strategic framework aimed at reducing Saudi Arabia’s dependence on oil, diversifying its economy, and developing public service sectors such as health, education, infrastructure, recreation, and tourism (12). This ambitious plan includes significant investments in the healthcare system to improve accessibility, quality of care, and health outcomes, aligning with the United Nations Sustainable Development Goal 3 (SDG 3), which aims to ensure healthy lives and promote well-being for all at all ages, including mental health and non-communicable diseases (NCDs) (13–15).

Moreover, the World Health Organization (WHO) Eastern Mediterranean Regional Office (EMRO) Mental Health Atlas provides a comprehensive overview of the mental health systems in the region, highlighting the need for improved mental health services and policies in line with Vision 2030’s goals (16). These efforts are crucial for addressing the significant burden of mental health disorders in the region and enhancing the integration of mental health into primary healthcare settings.

Additionally, the impact of global phenomena, such as the COVID-19 pandemic, has underscored the importance of mental health awareness and care. The pandemic has not only heightened general stress levels but also brought mental health issues to the forefront of public discourse, thereby influencing perceptions and acceptance of mental health challenges and services (17). The integration of digital health technologies within Saudi Arabia marks a pivotal moment for enhancing mental health literacy and accessibility to services. Supported by the digital transformation goals of Vision 2030, telepsychiatry and mobile health applications emerge as key tools in dismantling cultural barriers and reducing stigma around mental health. This digital shift opens up new avenues for public education, early detection of mental health issues, and the provision of counseling services remotely, significantly transforming societal engagement with mental health care towards more contemporary methods (18).

Gender differences in perceptions of mental disorder causes and treatments were also notable. Females were more likely to attribute mental disorders to psychological trauma, domestic violence, and family history, reflecting broader themes of mental health literacy and help-seeking behaviors observed in the Arab world (19). Understanding how gender influences perceptions of mental health can provide deeper insights into tailored interventions, as gender-specific attitudes and beliefs can significantly impact help-seeking behaviors and treatment preferences. This suggests a need for gender-sensitive approaches in mental health education and intervention programs. These insights have implications for public health policies, the design of mental health services, and educational campaigns aimed at reducing stigma and improving mental health literacy (20).

This study seeks to explore the general population’s perceptions in Saudi Arabia regarding the causes, treatments, and prevention of mental disorders. Specifically, it aims to assess how cultural and religious beliefs influence these perceptions and how they compare to the scientific understanding of mental health. The findings have important implications for public health policies, the design of mental health services, and educational campaigns aimed at reducing stigma and improving mental health literacy.

## 2 Materials and methods

### 2.1 Design

A national cross-sectional screening study was employed in this study. To ensure consistency and allow for comparison with previous years, we adopted the Saudi Arabia Mental Health Surveillance System (MHSS) protocol. The MHSS was evaluated for consistency and sensitivity in a separate study. The findings for 2020 were published in a peer-reviewed journal, demonstrating its robust methodological properties (21). The MHSS protocol, which includes detailed procedures for data collection, sampling, and analysis, has been validated through peer-reviewed research. These studies confirmed the protocol’s high sensitivity and consistency in accurately identifying mental health issues within the Saudi population. Thus, the MHSS protocol is the most rigorous reference with recent historical results available for mental health screening in Saudi Arabia (22).

### 2.2 Participants

The current research employed the proportional quota sampling method to ensure an equitable representation of participants, stratified by age, gender, and region. This approach resulted in a quota of 4 strata for each region, potentially enhancing sample diversity and mitigating non-probability sampling biases. The sample size was determined according to the necessary depth of sub-analysis, aimed at comparing age and gender groups across regions, with a medium effect size of 0.25, 80% power, and 95% confidence interval (22). Thus, each quota required 124 participants, resulting in a total sample of 4,547 participants.

Once the quota sample was achieved, participants with similar characteristics were not eligible to participate in the study and were excluded automatically via the ZdataCloud data collection system (23). This project used the proportional quota sampling technique to generate equal quotas/strata based on age, gender, and regions. Quota sampling operates as an automated procedure devoid of human intervention, wherein the sampling process is autonomously managed by the data collection system. This feature serves to eradicate the possibility of human sampling bias and enhances the overall quality of sampling (22).

### 2.3 Instruments

The data collection form included general demographic variables such as age, gender, region, and educational level. Additional variables included previous diagnosis of a mental disorder, living with someone with a mental disorder, and working in the healthcare sector. The instrument consisted of questions measuring perceptions, treatments, and interventions toward mental health disorders. A team of mental health and public health experts conducted a focus group session to explore common beliefs in Saudi society about the causes, treatment, and prevention of mental illness. These beliefs were then refined and confirmed through further studies.

The tool was developed and validated through a rigorous multi-stage process adhering to scientific standards. Initially, an expert panel reviewed and refined the tool to ensure relevance and comprehensiveness. This was followed by a focus group for linguistic validation, enhancing the phrasing and clarity of the 30 statements. The validation process compared a Likert scale and a Yes/No answer format to confirm perceptions, demonstrating very good internal consistency and reliability. The Cronbach’s Alpha values were as follows: perceived causes of mental illness (0.770, 10 items), perceived mental illness treatments (0.765, 13 items), and perceived mental disorder prevention (0.727, 7 items). Additionally, the tool showed high internal consistency with an overall Cronbach’s Alpha exceeding 0.8 and an Intraclass Correlation surpassing 0.85.

The Yes/No answer format was found to be superior to the Likert scale based on linguistic validation and focus group feedback, ensuring the tool’s reliability and validity in assessing perceptions of mental health disorders.

### 2.4 Procedures & data analyses

#### 2.4.1 Procedures

The participants were selected via a random phone number list generated from the Sharik Association for Health Research, a research participants’ database. The Sharik database is a database of individuals interested in participating in health research that currently has more than 64,000 potential participants and grows on a daily basis, covering the 13 administrative regions of Saudi Arabia (24). Participants were contacted via SMS to inform them of their eligibility for participation. Data collectors then conducted phone interviews using the ZDataCloud data collection system, which controlled sample distribution and ensured all questions were answered for successful submission (25). The interview or self-reporting process took approximately 13 minutes to complete. All data collectors received training on research ethics, data quality, and the use of the electronic data collection system, as well as specific training on administering the screening tools.

A subset comprising ten percent of the subjects underwent testing with the data collection tools during a pilot study. These participants were subsequently excluded from the primary research study. The pilot study served to evaluate the suitability of the research instruments and estimate the time required to administer them. Insights gathered from the pilot study were instrumental in refining the tools, involving corrections and additions to certain items.

#### 2.4.2 Data analysis

Data analysis was conducted using the Statistical Package for the Social Sciences (SPSS), Version 23 for Windows. Categorical measurements were represented using numbers and percentages, while numerical measurements were presented as mean and standard deviation (SD). The Monte Carlo chi-squared test was employed to assess potential relationships between the demographic characteristics of participants and their perceptions regarding the causes, prevention, and treatment of mental illnesses. Additionally, Cramer’s V was used to measure the strength of association between categorical variables. A multivariate logistic regression analysis was used to examine the relationships between demographic variables (age, gender, education level, income, marital status, history of mental health diagnosis, living with someone with a mental disorder, friendship with someone diagnosed with a mental disorder, and healthcare-related job) and the perceptions of mental disorder causes, treatments, and prevention strategies. The p-value threshold for determining statistical significance was set at 0.05.

### 2.5 Ethical considerations

The study was performed in accordance with the Declaration of Helsinki and followed Saudi Arabia Research Ethics Standards. Verbal consent was obtained at the beginning of the phone interview. All obtained data were de-identified to prevent the identification of individual participants. Ethics approval was granted by the Sharik Association for Research and Studies review board (IRB number: 09/2023).

## 3 Results

**Table 1** presents demographic and personal characteristics of the study population, consisting of 4547 participants. The distribution of sex is almost equal, with females slightly outnumbering males (50.1% vs. 49.9%). In terms of education, a majority of participants (55.0%) hold a bachelor’s degree or higher. The age group distribution shows a higher concentration of younger adults, with the largest proportion in the 20–29 age group (34.0%), followed by the 40–49 (22.9%) and 30–39 (21.9%) age groups. Regarding income, the most common category is an income of more than 5000 SAR/month, accounting for 45.2% of participants, while 33.2% reported an unstable monthly income. The participants were almost evenly split between single (49.7%) and married (50.3%) statuses. A small proportion of the sample reported a history of mental health diagnosis and treatment (6.0%), and a similar small percentage live with (13.5%) or are friends with (14.1%) someone diagnosed with mental illness. Finally, 16.2% of participants are employed in healthcare-related jobs.

**Table 1:** Demographic characteristics of study participants.

Variable		Proportion, n (%)
Sex	Male	2268 (49.9)
	Female	2279 (50.1)
Education Level	Less than bachelor’s degree	2047 (45.0)
	Bachelor’s degree or above	2500 (55.0)
Age Groups	18–19	226 (5.0)
	20–29	1544 (34.0)
	30–39	994 (21.9)
	40–49	1039 (22.9)
	50–59	535 (11.8)
	60+	209 (4.6)
Income Level	Unstable monthly income	1508 (33.2)
	Less than 5000 SAR/Month	982 (21.6)
	More than 5000 SAR/Month	2057 (45.2)
Current Marital Status	Single	2258 (49.7)
	Married	2289 (50.3)
Mental Health Diagnosis & treatment History	No	4274 (94.0)
	Yes	273 (6.0)
Living with a person diagnosed with mental illness	No	3933 (86.5)
	Yes	614 (13.5)
Friend with a person diagnosed with mental illness	No	3908 (85.9)
	Yes	639 (14.1)
Healthcare-related job	No	3809 (83.8)
	Yes	738 (16.2)

**Table 2** shows respondents’ perceptions of various factors contributing to mental disorders causes. A significant majority, 78.36%, believe psychological trauma is a cause, making it the most acknowledged factor. Drug and alcohol abuse (72.88%) and domestic violence and sexual harassment (69.39%) are also widely recognized as contributing factors. Over half of the respondents attribute mental disorders to loneliness and social isolation (64.06%), family history and genetics (62.11%), and lack of religious scruples (51.90%). Less than half, however, see chronic diseases (48.30%), poverty (38.66%), envy and the evil eye (35.89%), and magic (34.26%) as causes. These findings highlight a diverse range of beliefs about the etiology of mental disorders, with psychological trauma, substance abuse, and domestic violence seen as the leading causes by the respondents.

**Table 2:** respondents’ agreement with statements on perceived causes of mental disorders.

Statement	Yes n (%)	No n (%)
I think that the causes of mental disorders are due to psychological trauma.	3563 (78.36%)	984 (21.64%)
I think that the causes of mental disorders are due to drug and alcohol abuse.	3314 (72.88%)	1233 (27.12%)
I think that the causes of mental disorders are due to domestic violence and sexual harassment.	3155 (69.39%)	1392 (30.61%)
I think that the causes of mental disorders are due to loneliness and social isolation.	2913 (64.06%)	1634 (35.94%)
I think that the causes of mental disorders are due to family history and genetics.	2824 (62.11%)	1723 (37.89%)
I think that the causes of mental disorders are due to lack of religious scruples.	2360 (51.90%)	2187 (48.10%)
I think that the causes of mental disorders are due to chronic and incurable diseases.	2196 (48.30%)	2351 (51.70%)
I think that the causes of mental disorders are due to poverty and destitution.	1758 (38.66%)	2789 (61.34%)
I think that the causes of mental disorders are due to envy and the evil eye.	1632 (35.89%)	2915 (64.11%)
I think that the causes of mental disorders are due to magic.	1558 (34.26%)	2989 (65.74%)

**Table 3** indicates a strong preference among respondents for non-pharmacological psychotherapy (74.97%) and drug therapy (71.08%) as effective treatments for mental dis-orders. Additionally, a notable proportion of participants endorse spiritual and religious practices, such as the Sun-nah of the Prophet and reading the Holy Quran (58.24%), and Roquia in the Qur’an (56.74%), as viable approaches. Conversely, alternative therapies like cupping, energy healing, and neuro-programming therapy receive considerably less support, suggesting a greater reliance on established medical treatments and certain traditional practices in the perception of mental health treatment effectiveness.

**Table 3:** Respondents’ agreement with statements on perceived mental illness treatments.

Statement	Yes n (%)	No n (%)
I believe that mental disorders can be treated through drug therapy.	3232 (71.08%)	1315 (28.92%)
I believe that mental disorders can be treated through non-pharmacological psychotherapy.	3409 (74.97%)	1138 (25.03%)
I think that mental disorders can be treated through Roquia in the Qur’an.	2580 (56.74%)	1967 (43.26%)
I think that mental disorders can be treated through cupping.	913 (20.08%)	3634 (79.92%)
I believe that mental disorders can be treated through herbs and supplements.	1100 (24.19%)	3447 (75.81%)
I think that mental disorders can be treated through the Sunnah of the Prophet and reading the Holy Quran.	2648 (58.24%)	1899 (41.76%)
I believe that mental disorders can be treated through energy healing e.g. Reiki or Qigong.	666 (14.65%)	3881 (85.35%)
I believe that mental disorders can be treated through neuroprogramming therapy.	745 (16.38%)	3802 (83.62%)
I believe that mental disorders can be treated through yoga and relaxation exercises.	1364 (30.00%)	3183 (70.00%)
I believe that mental disorders can be treated through sessions with life coaches.	1618 (35.58%)	2929 (64.42%)
I believe that mental disorders can be treated through sports and walking.	2436 (53.57%)	2111 (46.43%)
I believe that mental disorders can be treated by talking to a friend or family member.	2330 (51.24%)	2217 (48.76%)
I believe that mental disorders can be treated through self-development courses.	1702 (37.43%)	2845 (62.57%)

**Table 4** reveals the perceptions of respondents on potential preventative measures for mental disorders. A significant majority, 80.4%, believe that increasing awareness of mental health is a key preventive strategy. Positive social relationships are also highly valued, with 70.6% agreeing that they can prevent mental disorders. Other notable measures include increasing physical activity (66.3%), following the instructions of the Sunnah and reading the Holy Quran (66.9%), and talking to a mental health professional (58.4%). While relaxation and meditation are seen as beneficial by more than half of the respondents (56.2%), self-development courses are considered less effective, with only 43.0% in agreement. These results indicate a strong belief in the importance of awareness, social support, physical health, and spiritual practices in preventing mental disorders.

**Table 4:** respondents’ agreement with statements on perceived mental disorders prevention.

Statement	Yes n (%)	No n (%)
I believe that mental disorders can be prevented by increasing awareness of mental health.	3657 (80.4%)	890 (19.6%)
I believe that mental disorders can be prevented by increasing your positive social relationships.	3209 (70.6%)	1338 (29.4%)
I believe that mental disorders can be prevented by increasing the time for relaxation and meditation.	2557 (56.2%)	1990 (43.8%)
I believe that mental disorders can be prevented through the instructions of the Sunnah and reading the Holy Quran.	3042 (66.9%)	1505 (33.1%)
I believe that mental disorders can be prevented by talking to a mental health professional.	2655 (58.4%)	1892 (41.6%)
I believe that mental disorders can be prevented through self-development courses.	1957 (43.0%)	2590 (57.0%)
I believe that mental disorders could be prevented by increasing physical activity, improving physical fitness and walking.	3016 (66.3%)	1531 (33.7%)

**Table 5**, the analysis of respondents’ views on the causes of mental disorders by gender reveals significant differences in several areas. Females are more likely than males to attribute mental disorders to psychological trauma, domestic violence, and family history, with statistically significant differences (p < 0.001). In contrast, beliefs about the role of drug and alcohol abuse, lack of religious scruples, and magic show no significant gender differences. Notably, there are moderate gender differences in the perception of poverty and envy as causes. This indicates varied perspectives between males and females on certain factors contributing to mental illness.

**Table 5:** Respondents’ agreement with statements on perceived causes of mental illness by gender.

	Male		Female		Chi-Square (p value)	Cramér’s V
Statement	Yes	No	Yes	No		
I think that the causes of mental disorders are due to (psychological trauma)	1679 (74.0%)	589 (26.0%)	1884 (82.7%)	395 (17.3%)	50.0 (<0.001)	0.105
I think that the causes of mental disorders are due to (drug and alcohol abuse)	1639 (72.3%)	629 (27.7)	1675 (73.5%)	604 (26.5%)	0.87 (0.351)	0.014
I think that the causes of mental disorders are due to (domestic violence and sexual harassment)	1468 (64.7%)	800 (35.3%)	1687 (74.0%)	592 (26.0%)	46.2 (<0.001)	0.101
I think that the causes of mental disorders are due to (loneliness and social isolation)	1429 (63.0%)	839 (37.0%)	1484 (65.1%)	795 (34.9%)	2.1 (0.138)	0.022
I think that the causes of mental disorders are due to (family history and genetics)	1334 (58.8%)	934 (41.2%)	1490 (65.4%)	789 (34.6%)	20.7 (<0.001)	0.068
I think that the causes of mental disorders are due to (lack of religious scruples)	1175 (51.8%)	1093 (48.2%)	1185 (52.0%)	1094 (48.0%)	0.16 (0.899)	0.003
I think that the causes of mental disorders are due to (chronic and incurable diseases)	1124 (49.6%)	1144 (50.4%)	1072 (47.0%)	1207 (53.0%)	2.8 (0.089)	0.025
I think that the causes of mental disorders are due to (poverty and destitution)	933 (41.1%)	1335 (58.9%)	825 (36.2%)	1454 (63.8%)	11.6 (0.001)	0.050
I think that the causes of mental disorders are due to (envy and the evil eye)	782 (34.5%)	1486 (65.5%)	850 (37.3%)	1429 (62.7%)	3.9 (0.048)	0.029
I think that the causes of mental disorders are due to (magic)	760 (33.5%)	1508 (66.5%)	798 (35.0)	1481 (65.0%)	1.1 (0.285)	0.016

**Table 6**, provides insights into gender differences in beliefs about the effectiveness of various treatments for mental disorders. Notably, there are no significant gender differences in perceptions of drug therapy, non-pharmacological psychotherapy, Roquia in the Qur’an, cupping, herbs and supplements, energy healing, and neuro-programming therapy as treatments (p > 0.05). However, significant gender differences emerge in views on yoga and relaxation exercises, sessions with life coaches, physical activity, talking to a friend or family member, and self-development courses (p < 0.001), with females more likely to view these as effective treatments. These results suggest respondents’ agreement with statements on perceived mental illness prevention by Gender.

**Table 6:** Respondents’ agreement with statements on perceived mental illness treatments by Gender.

	Male		Female		Chi-Square (p value)
Statement	Yes	No	Yes	No	
I believe that mental disorders can be treated through (drug therapy).	1594 (70.3%)	674 (29.7%)	1638 (71.9%)	641 (28.1%)	1.4 (0.237)
I believe that mental disorders can be treated through (non-pharmacological psychotherapy)	1679 (74.0%)	589 (26.0%)	1730 (75.9%)	549 (24.1%)	2.1 (0.143)
I think that mental disorders can be treated through (Roquia in the Qur’an)	1288 (56.8%)	980 (43.2%)	1292 (56.7%)	987 (43.3%)	0.005 (0.947)
I think that mental disorders can be treated through (cupping)	449 (19.8%)	1819 (80.2%)	464 (20.4%)	1815 (79.6%)	0.2 (0.636)
I believe that mental disorders can be treated through (herbs and supplements)	532 (23.5%)	1736 (76.5%)	568 (24.9%)	1711 (75.1%)	1.3 (0.248)
I think that mental disorders can be treated through (the Sunnah of the Prophet and reading the Holy Quran)	1291 (56.9%)	977 (43.1%)	1357 (59.5%)	922 (40.5%)	3.2 (0.073)
I believe that mental disorders can be treated through (energy healing e.g. Reiki or Qigong)	325 (14.3%)	1943 (85.7%)	341 (15.0%)	1938 (85.0%)	0.3 (0.546)
I believe that mental disorders can be treated through (neuroprogramming therapy)	371 (16.4%)	1897 (83.6%)	374 (16.4%)	1905 (83.6%)	0.002 (0.962)
I believe that mental disorders can be treated through (yoga and relaxation exercises)	571 (25.2%)	1697 (74.8%)	793 (34.8%)	1486 (65.2%)	50.1 (<0.001)
I believe that mental disorders can be treated through (sessions with life coaches)	720 (31.7%)	1548 (68.3%)	898 (39.4%)	1381 (60.6%)	29.1 (<0.001)
I believe that mental disorders can be treated through (sports and walking)	1133 (50.0%)	1135 (50.0%)	1303 (57.2%)	976 (42.8%)	23.8 (<0.001)
I believe that mental disorders can be treated by (talking to a friend or family member)	1061 (46.8%)	1207 (53.2%)	1269 (55.7%)	1010 (44.3%)	36.0 (<0.001)
I believe that mental disorders can be treated through (self-development courses)	757 (33.4%)	1511 (66.6%)	945 (41.5%)	1334 (58.5%)	31.7 (<0.001)

**Table 7** shows the multivariate logistic regression analysis highlights significant associations between various demographic variables and perceptions of mental disorder causes, specifically psychological trauma, drug and alcohol abuse, and domestic violence and sexual harassment. Females are more likely than males to perceive these factors as causes. Higher education levels are significantly associated with perceiving domestic violence and sexual harassment as causes, while age is associated with a decreased likelihood of attributing mental disorders to drug and alcohol abuse. Marital status shows a significant association with perceiving drug and alcohol abuse as a cause. Participants with a mental health diagnosis and those living with or being friends with someone diagnosed with a mental disorder are more likely to attribute mental disorders to these causes. Additionally, working in a healthcare-related job is significantly associated with perceiving psychological trauma and domestic violence and sexual harassment as causes.

**Table 7:** Multivariate logistic regression analysis for perceived causes of mental disorders.

Variable	Psychological Trauma OR (95% CI)	Drug and Alcohol Abuse OR (95% CI)	Domestic Violence and Sexual Harassment OR (95% CI)
**Sex (Female vs. Male)**	1.15 (1.05–1.26)*	1.22 (1.10–1.35)*	1.30 (1.18–1.44)*
**Education (Higher vs. Lower)**	1.05 (0.96–1.15)	1.08 (0.98–1.19)	1.12 (1.02–1.23)*
**Age**	1.01 (0.99–1.02)	0.98 (0.96–0.99)*	1.00 (0.98–1.02)
**Income**	0.98 (0.89–1.08)	0.95 (0.86–1.06)	0.97 (0.88–1.08)
**Marital Status (Married vs. Single)**	1.04 (0.94–1.14)	1.11 (1.00–1.23)*	1.07 (0.97–1.19)
**Mental Health Diagnosis**	1.21 (1.01–1.45)*	1.25 (1.04–1.50)*	1.19 (0.99–1.43)
**Living with Mental Disorder**	1.11 (0.97–1.28)	1.18 (1.03–1.36)*	1.20 (1.04–1.38)*
**Friend with Mental Disorder**	1.10 (0.97–1.25)	1.09 (0.96–1.24)	1.14 (1.00–1.30)*
**Healthcare-related Job**	1.15 (1.01–1.31)*	1.12 (0.98–1.28)	1.18 (1.03–1.35)*

*p < 0.05.

**Table 8** demonstrates the multivariate logistic regression analysis for perceived mental illness treatments reveals significant associations between various demographic variables and the endorsement of drug therapy, non-pharmacological psychotherapy, and Roquia in the Qur’an as effective treatments. Females are more likely than males to endorse all three treatments. Higher education levels do not show significant associations with any of the treatments. Older age is associated with a decreased likelihood of endorsing non-pharmacological psychotherapy. Participants with a mental health diagnosis and those living with or being friends with someone diagnosed with a mental disorder are more likely to endorse non-pharmacological psychotherapy and Roquia in the Qur’an. Working in a healthcare-related job is significantly associated with endorsing drug therapy and non-pharmacological psychotherapy.

**Table 8:** Multivariate logistic regression analysis for perceived mental illness treatments.

Variable	Drug Therapy OR (95% CI)	Non-Pharmacological Psychotherapy OR (95% CI)	Roquia in the Qur’an OR (95% CI)
**Sex (Female vs. Male)**	1.10 (1.00–1.21)*	1.12 (1.02–1.24)*	1.15 (1.04–1.27)*
**Education (Higher vs. Lower)**	1.05 (0.96–1.15)	1.07 (0.97–1.18)	1.08 (0.98–1.20)
**Age**	1.01 (0.99–1.02)	0.98 (0.96–0.99)*	1.00 (0.98–1.02)
**Income**	0.99 (0.90–1.09)	0.97 (0.87–1.08)	0.95 (0.86–1.06)
**Marital Status (Married vs. Single)**	1.03 (0.94–1.14)	1.09 (0.98–1.21)	1.05 (0.94–1.17)
**Mental Health Diagnosis**	1.18 (0.99–1.41)	1.22 (1.02–1.46)*	1.24 (1.03–1.49)*
**Living with Mental Disorder**	1.12 (0.98–1.28)	1.17 (1.03–1.35)*	1.19 (1.04–1.36)*
**Friend with Mental Disorder**	1.09 (0.96–1.24)	1.10 (0.97–1.25)	1.13 (1.00–1.29)*
**Healthcare-related Job**	1.14 (1.00–1.31)*	1.15 (1.00–1.33)*	1.12 (0.98–1.28)

*p < 0.05.

**Table 9** revealed the multivariate logistic regression analysis for perceived mental disorder prevention indicates significant associations between demographic variables and beliefs in the effectiveness of increasing awareness, positive social relationships, and physical activity as preventive measures. Females are more likely than males to endorse all three prevention strategies. Higher education levels do not show significant associations with any of the preventive measures. Older age is associated with a decreased likelihood of endorsing positive social relationships as a prevention strategy. Participants with a mental health diagnosis, as well as those living with someone diagnosed with a mental disorder, are more likely to endorse positive social relationships and physical activity as preventive measures.

**Table 9:** Multivariate logistic regression analysis for perceived mental disorder Prevention.

Variable	Awareness OR (95% CI)	Positive Social Relationships OR (95% CI)	Physical Activity OR (95% CI)
**Sex (Female vs. Male)**	1.12 (1.01–1.24)*	1.14 (1.03–1.27)*	1.15 (1.04–1.28)*
**Education (Higher vs. Lower)**	1.06 (0.96–1.17)	1.09 (0.98–1.21)	1.08 (0.97–1.20)
**Age**	1.00 (0.99–1.02)	0.98 (0.96–0.99)*	1.00 (0.98–1.02)
**Income**	0.98 (0.88–1.08)	0.97 (0.87–1.08)	0.95 (0.85–1.05)
**Marital Status (Married vs. Single)**	1.04 (0.94–1.15)	1.08 (0.97–1.21)	1.05 (0.94–1.18)
**Mental Health Diagnosis**	1.17 (0.98–1.39)	1.20 (1.00–1.43)*	1.23 (1.02–1.49)*
**Living with Mental Disorder**	1.13 (0.99–1.29)	1.16 (1.02–1.32)*	1.19 (1.04–1.36)*
**Friend with Mental Disorder**	1.08 (0.95–1.23)	1.09 (0.96–1.24)	1.12 (0.99–1.28)
**Healthcare-related Job**	1.13 (0.98–1.30)	1.15 (0.99–1.34)	1.12 (0.97–1.29)

*p < 0.05.

## 4 Discussion

The study conducted on perceptions of mental health in Saudi Arabia reveals a significant trend in societal beliefs. In our study, 86.5% of participants reported not living with a person diagnosed with mental illness, while 13.5% did and 14.1% are friends with someone diagnosed with mental illness. Contrastingly, the study found that 36% of participants reported having at least one relative with mental illness, while 64% were unsure. This significant difference in awareness and personal connection to mental illness between the two studies may reflect varying levels of mental health stigma, cultural openness about mental health issues (26).

The study results indicate that a majority of respondents identify psychological trauma (78.36%), drug and alcohol abuse (72.88%), and domestic violence and sexual harassment (69.39%) as key causes of mental disorders. These findings align with research from other Gulf countries and broader Middle Eastern contexts, where similar factors are frequently recognized as significant contributors to mental health issues. The recognition of these causes highlights an awareness of both personal and societal contributors to mental health, which is crucial for developing effective intervention strategies (7). These findings also are consistent with studies conducted in other Gulf and Arab Muslim countries. For example, a study in Kuwait conducted by Al-Haddad et al. (27), found that psychological trauma and substance abuse were perceived as major causes of mental disorders, with a significant portion of the population attributing mental health issues to familial and social stressors (27).

Similarly, research in the United Arab Emirates conducted by Eapen et al. (28) highlighted that domestic violence and genetic predispositions were commonly believed to contribute to mental health problems. In Oman, a study about perception of mental illness in Oman revealed that substance abuse and social isolation were seen as primary factors leading to mental disorders. These parallels underscore the widespread recognition of these causes across different cultural contexts within the Gulf and broader Arab region, emphasizing the need for region-specific mental health interventions that address these commonly perceived causes (28–30).

In the context of study’s findings on the risk of depression based on participants’ beliefs about perceived mental disorder prevention, the study highlight that females are more likely to attribute mental disorders to factors such as psychological trauma, domestic violence, and family history, align with the broader themes of mental health literacy and help-seeking behaviors observed in the Arab world. The systematic review on MHL in the Gulf Cooperation Council countries underscores the importance of understanding mental disorders and their management or prevention. This is particularly relevant in overcoming barriers like stigma and limited access to mental health care, which the study also seems to touch upon through its exploration of perceived prevention methods (31).

Specific mental health disorders, such as depression and suicide, also warrant attention in the Saudi context. Our study found that a significant majority of respondents (78.36%) identify psychological trauma as a primary cause of mental disorders, and there is a strong belief in the importance of increasing awareness of mental health (80.4%) as a key preventive strategy. These findings highlight the need for effective mental health education and intervention strategies. While suicide is considered haram (forbidden) in Islam, understanding the cultural and religious nuances surrounding mental health, as discussed by Padela et al. (32), can aid in developing these strategies. Addressing issues like depression and suicide through the lens of Islamic teachings can enhance the acceptance and effectiveness of mental health care in Saudi Arabia. The integration of religious beliefs with modern mental health practices, as reflected in the respondents’ support for spiritual practices like reading the Holy Quran (58.24%) and Roquia (56.74%), underscores the potential for a more comprehensive and culturally sensitive approach to mental health care (32).

Historically, Islamic scholarship has significantly contributed to the understanding and treatment of mental health. This historical context provides a rich foundation for contemporary mental health practices in Muslim societies, including Saudi Arabia. Our study’s findings, which show a strong endorsement for non-pharmacological psychotherapy (74.97%) and drug therapy (71.08%), along with substantial support for spiritual practices such as the Sunnah of the Prophet and reading the Holy Quran (58.24%), and Roquia in the Qur’an (56.74%), reflect this integration of medical and spiritual approaches. These results align with the work of contemporary scholars like Rania Awad, who advocate for the integration of Islamic psychology into mental health care to enhance its relevance and effectiveness for Muslim. The significant role of religious practices in mental health treatment preferences among our respondents underscores the importance of culturally sensitive and religiously congruent mental health interventions in Saudi Arabia (33, 34).

Regarding perception of the participants for treatment methods for the mental illness, study’s findings revealed a strong preference for both non-pharmacological psychotherapy and drug therapy among respondents. Interestingly, a significant proportion also endorses spiritual practices like the Sunnah of the Prophet and Roquia in the Qur’an. These results consistent with other study conducted at Saudi Arabia about mental health and revealed that the stigma associated with mental illness often leads individuals to seek help from religious leaders rather than mental health professionals. This underscores the importance of incorporating spiritual care into mental health services to enhance their acceptance and effectiveness (4).

Similarly, a study in the United Arab Emirates (UAE) indicated that while there is a growing acceptance of psychiatric medications and psychotherapy, a significant number of people still rely on traditional and spiritual healers. The coexistence of modern and traditional beliefs in treating mental health problems suggests that healthcare providers need to consider these cultural factors when developing treatment plans (27). Also these results are approved by a study conducted in Oman, it was found that there is a substantial reliance on spiritual and religious practices for treating mental health issues. The participants often preferred combining modern medical treatments with traditional religious practices, such as prayer and recitation of the Qur’an (9).

Moreover, the integration of Islamic psychology into mental health care can bridge the gap between traditional beliefs and modern practices. Abdallah Rothman, Malik Badri, and Rania Awaad have extensively written on this integration, emphasizing how Islamic teachings and psychology can work together to provide holistic mental health care. Rothman highlights the importance of incorporating faith-based principles into therapy to enhance the therapeutic process for Muslim patients. Badri’s work underscores the necessity of understanding the spiritual dimensions of mental health from an Islamic perspective, which can lead to better acceptance and outcomes of psychiatric interventions. Awaad’s contributions focus on how religious coping mechanisms and Islamic practices can be effectively used alongside conventional treatments to address mental health issues within Muslim communities. By including these perspectives, our study emphasizes the need for mental health strategies that are not only scientifically sound but also culturally and religiously sensitive, thus improving the overall mental health landscape in Saudi Arabia (33, 35, 36).

Preventive measures for mental disorders, as perceived by the respondents, emphasize the importance of increasing awareness (80.4%) and fostering positive social relationships (70.6%). These preventive strategies resonate with findings from studies in other Muslim-majority countries, where community support and social cohesion are seen as protective factors against mental illness. Additionally, the emphasis on following the instructions of the Sunnah and reading the Holy Quran (66.9%) highlights the role of religious teachings in promoting mental well-being (7).

The study results’ offers insightful data that can be contrasted with the broader regional findings presented in the Janssen report “Mental Health in the Middle East: Measuring Progress towards Integrated, Accessible, and Equitable Mental Health.” The present study, with its focus on individual and societal beliefs about mental disorders, reveals perceptions that are crucial for shaping tailored mental health policies and interventions. This approach aligns with the World Health Organization’s definition of mental health as a state enabling individuals to cope with life’s stresses, learn, work well, and contribute to their community. Meanwhile, the Janssen report provides a macro-level view, assessing the prevalence, burden, and policy responses to mental health across several MENA countries, using metrics such as disability-adjusted life years (DALYs) and mortality from self-harm. The comparison of these studies underscores the complexity of mental health challenges in the region and highlights the importance of culturally sensitive, comprehensive strategies that integrate mental health services with primary care, improve mental health literacy, and ensure accessible care (37).

Finally, these study adds significant insights into mental health perceptions in the region. It highlights the predominant belief among respondents that psychological trauma, drug and alcohol abuse, and domestic violence are key causes of mental disorders. Studying dominant and common beliefs is important, not to change them, but to integrate them when they are not fundamentally incompatible with the principles of mental health. For instance, the relationship between religious beliefs and mental health underscores the importance of integrating religious and psychological aspects. Religious beliefs are widespread, and the results indicate that some have a positive correlation with the risk of depression. A similar conclusion can be drawn regarding the relationship between social beliefs and mental health.

The study recommends a comprehensive approach to enhance mental health awareness and treatment in Saudi Arabia. Key strategies include collaborating with Islamic Studies, Sharia, and Psychology faculties to address misconceptions and harmonize understanding of mental health. Training mosque imams and prayer leaders, in partnership with the Ministry of Islamic Affairs, is advised to correct misconceptions and raise awareness. Coordinated efforts by the Quality-of-Life Program, the Sports Ministry, and the Sports for All Program are highlighted to promote mental health, particularly among men, through sports and effective communication. Special awareness programs are suggested for individuals with less than a bachelor’s degree, in collaboration with the Ministry of Human Resources, focusing on the causes, treatment, and prevention of mental illness. Targeted programs are also recommended for those at high risk of depression or anxiety, involving ongoing assessments and tailored interventions. Finally, a reassessment of these initiatives after two years is suggested to measure their impact and ensure their effectiveness in changing beliefs and practices related to mental health.

Future studies should focus on longitudinal research to observe changes in mental health perceptions and behaviors over time, particularly in response to ongoing mental health awareness campaigns and interventions. Additionally, research could explore the effectiveness of integrating religious and spiritual practices into conventional mental health treatments to assess their impact on treatment outcomes and acceptance. Investigating the role of digital health technologies, such as telepsychiatry and mobile health applications, in improving access to mental health services and reducing stigma in Saudi Arabia would also be valuable. Furthermore, comparative studies across different regions within Saudi Arabia and other Gulf countries could provide deeper insights into regional variations in mental health perceptions and inform more tailored intervention strategies.

## 5 Limitations

Our research offers important insights into the subject, yet it comes with certain limitations. Firstly, due to its survey-based approach, there’s a possibility of self-reporting bias, where participants might not always provide accurate or truthful responses about their experiences or views. Furthermore, the cross-sectional design of our study captures only a single moment in time, which hampers our ability to establish cause and effect or observe trends over periods. Also, our study did not consider some variables that could have influenced the outcomes. Even though we conducted thorough training for interviewers, the possibility of interviewer bias remains a concern in our research.

## 6 Conclusions

The study revealed significant insights into the general population’s perceptions of mental health in Saudi Arabia, indicating a need for further awareness and education on mental health disorders, treatments, and prevention strategies. It also highlights the importance of integrating religious and social beliefs into mental health programs for better community engagement and effectiveness.

## Data availability statement

The original contributions presented in the study are included in the article/supplementary material. Further inquiries can be directed to the corresponding author.

## Ethics statement

The studies involving humans were approved by The ethics committee of the Sharik Association for Health Research approved this research project (Approval no. 2023-8), in accordance with national research ethics regulations. The studies were conducted in accordance with the local legislation and institutional requirements. The participants provided their written informed consent to participate in this study.

## Author contributions

MS: Writing – original draft, Writing – review & editing. AA: Writing – original draft, Writing – review & editing. NA: Writing – original draft, Writing – review & editing. MA: Writing – original draft, Writing – review & editing. RA: Writing – original draft, Writing – review & editing. NB: Writing – original draft, Writing – review & editing.
